# HDAC6 promotes sepsis development by impairing PHB1-mediated mitochondrial respiratory chain function

**DOI:** 10.18632/aging.102964

**Published:** 2020-03-28

**Authors:** Shi-dong Guo, Sheng-tao Yan, Wen Li, Hong Zhou, Jian-ping Yang, Yao Yao, Mei-jia Shen, Liu-wei Zhang, Hong-Bo Zhang, Li-Chao Sun

**Affiliations:** 1 Emergency Department of China-Japan Friendship Hospital, Beijing, China; 2 Surgical Intensive Care Unit of China-Japan Friendship Hospital, Beijing, China; 3 Department of Emergency, China Emergency General Hospital, Beijing, China; 4 Department of Physical Constitution and Health, Sport Science College, Beijing Sport University, Beijing, China

**Keywords:** HDAC6, PHB1, mitochondrial respiratory control rate, oxidative stress, CLP-induced sepsis

## Abstract

Objective: This study was aimed at investigating the regulation of mitochondrial function by histone deacetylase 6 (HDAC6) and the role of HDAC6 in the development and progression of sepsis.

Results: HDAC6 downregulated PHB1 and subsequently promoted the development of CLP-induced sepsis. Inhibition of HDAC6 significantly attenuated CLP-induced sepsis through inhibition of mitochondrial dysfunction and reduced oxidant production, thus protecting the rats from oxidative injury.

Conclusions: In this sepsis model, HDAC6 inhibits the expression and function of PHB1 and alters the function of the mitochondrial respiratory chain mediated by PHB1, thus enhancing the production of oxidants and increasing oxidative stress and thereby leading to severe oxidative injury in multiple organs.

Methods: The expression of HDAC6 and prohibitin 1 (PHB1) in humans and in a rat model of sepsis was measured by quantitative reverse-transcription PCR and western blotting. Sepsis induction by cecal ligation and puncture (CLP) was confirmed by histological analysis. Concentrations of different sepsis markers were measured by an enzyme-linked immunosorbent assay, and mitochondrial function was assessed via the mitochondrial respiratory control rate.

## INTRODUCTION

Sepsis is a systemic inflammatory response syndrome caused by infection and is considered to be the major cause of death among patients in intensive care units owing to its relatively high morbidity and mortality rate [1–3]. It can impair the function of multiple organs, e.g., it can cause lung, renal, and hepatic injury, thereby leading to multiple organ dysfunction syndrome [4, 5]. In the past decade, progress was made in the development of therapies for sepsis. Nonetheless, sepsis still has a high mortality rate. Therefore, investigation of the pathogenesis including the molecular mechanisms underlying sepsis is important for designing strategies for sepsis prevention and treatment.

Oxidative stress, caused by inflammatory responses, is a known consequence of sepsis [6, 7], and increased lipid [8] and protein oxidation have been observed in patients with sepsis [9]. Recently, studies have revealed that the production of reactive oxygen species (ROS) and mitochondrial dysfunction plays an essential role in the development of sepsis [6, 10]. Therefore, mitochondria-targeted antioxidant therapy may be an effective modality for patients with sepsis or septic shock.

HDAC6 is a member of the cytoplasmic class IIb histone deacetylase (HDAC) family. It deacetylates nonhistone proteins [11], including heat shock protein 90 (HSP90) [12], α-tubulin [13], and cortactin [14, 15]. Therefore, HDAC6 inhibits or stimulates multiple important biological processes, including degradation of misfolded proteins, stress granule formation, viral infection, immune synapse formation, cell spreading, and cell migration [11]. Additionally, one study suggests that inhibition of HDAC6 with a selective HDAC6 inhibitor, such as tubastatin A, alleviates several inflammatory and autoimmune diseases [16].

Prohibitin 1 (PHB1) is a ubiquitously expressed and highly conserved protein [17]. *PHB1* is located in chromosomal region 17q21, and encodes a 30 kDa protein that associates with prohibitin 2 (PHB2) to form 16–20-mer ringlike structures that perform chaperone or scaffolding activities within the mitochondria [18, 19]. It has been reported that PHB1 is essential for normal mitochondrial development, and that PHB1 deficiency in *Caenorhabditis elegans* is associated with inhibition of mitochondrial biogenesis and senescence [20, 21]. Additionally, PHB1 is involved in cell cycle control [22], differentiation [23], apoptosis [22], and senescence [21], thereby emerging as a central determinant of cell fate. Alteration in PHB1 levels is associated with pathologies, such as inflammation [24], obesity [25], autoimmunity [26], and cancer [27].

In the present study, we examined the effects of HDAC6 and PHB1 on the progression of sepsis. We also analyzed the correlations among HDAC6, PHB1, and mitochondrial dysfunction, and investigated their role in the development and progression of sepsis. These results could shed new light on a possible target for the diagnosis and treatment of sepsis.

## RESULTS

### Elevated HDAC6 and reduced PHB1 expression is associated with sepsis

**Figure 1:**
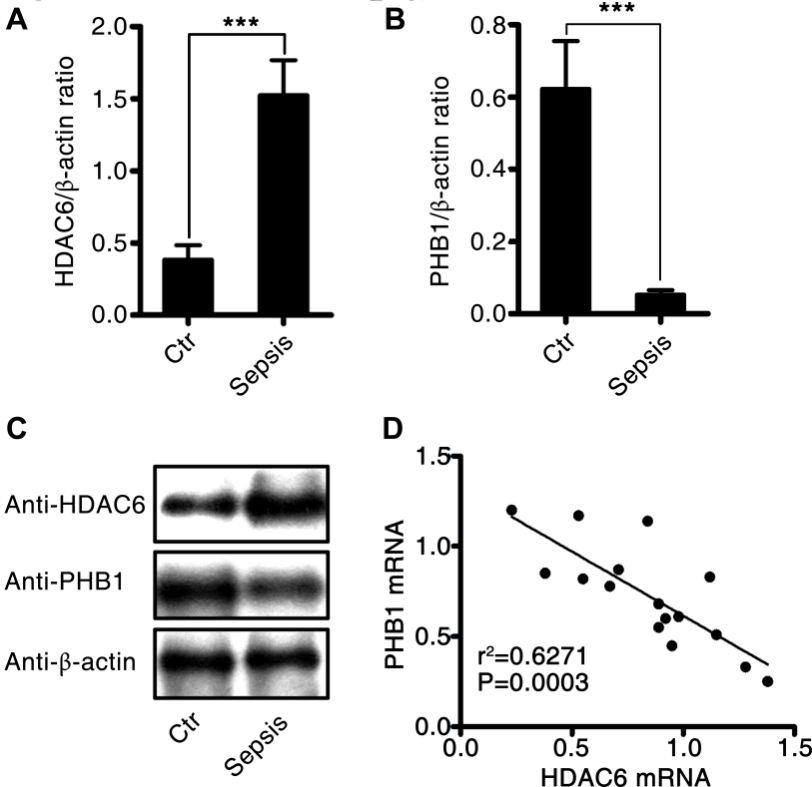
**Expression and correlation of HDAC6 and PHB1 in sepsis patients.** Human PBMCs were isolated from healthy control participants and patients with sepsis. HDAC6 and PHB1 mRNA and protein levels were measured by qPCR and western blotting, respectively. The linear correlation between HDAC6 and PHB1 expression was analyzed using the GraphPad Prism software. (**A**) *HDAC6* mRNA; (**B**) *PHB1* mRNA; (**C**) HDAC6 and PHB1 protein expression; (**D**) the correlation between HDAC6 and PHB1 expression. Results are expressed as the mean ± SEM. *P < 0.05; **P < 0.01; ***P < 0.001.

To investigate the effects of HDAC6 and PHB1 on the development of sepsis, we measured the expression of HDAC6 and PHB1 in peripheral blood mononuclear cells (PBMCs) derived from sepsis patients and healthy controls. *HDAC6* mRNA expression was higher in PBMCs from patients with sepsis than that in healthy control PBMCs (1.5 ± 0.25 vs 0.4 ± 0.1, P < 0.001; Figure 1A). Conversely, *PHB1* mRNA expression was lower in PBMCs from sepsis patients as compared with PBMCs from healthy controls (0.05 ± 0.024 vs 0.62 ± 0.15, P < 0.001; Figure 1B.). These results were consistent with the expression of HDAC6 and PHB1 proteins in PBMCs from sepsis patients and healthy control participants (Figure 1C). A negative linear correlation was observed between HDAC6 expression and that of PHB1 in PBMCs from sepsis patients (r^2^ = 0.6271, P = 0.0003; Figure 1D). Taken together, these results indicated that HDAC6 expression has a significant inverse correlation with PHB1 levels, and that it directly correlates with disease severity in patients with sepsis.

### Construction of a rat model of cecal ligation and puncture (CLP)-induced sepsis

**Figure 2:**
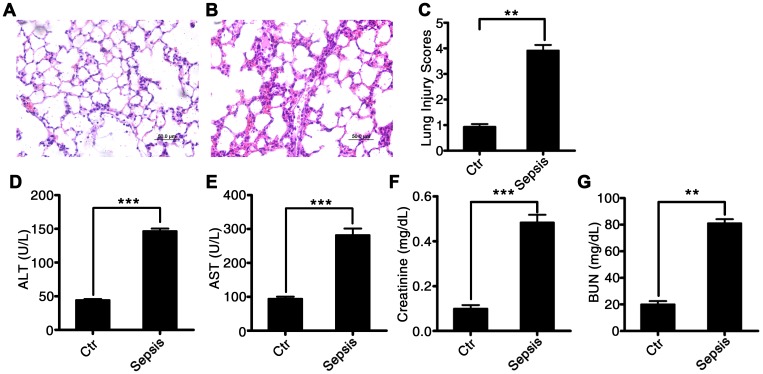
**Characterization of CLP-induced sepsis in rats.** Serum and tissue samples from rats with untreated CLP-induced sepsis, rats with Tri A–treated CLP sepsis, or healthy control rats. (**A**) Lung tissue from the control group of rats; (**B**) lung tissue from rats with CLP-induced sepsis; (**C**) lung injury index; (**D**) ALT activity; (**E**) AST activity; (**F**) creatinine concentration; and (**G**) BUN levels in the plasma from healthy control rats and CLP-induced sepsis rats. Results are expressed as the mean ± SEM. *P < 0.05; **P < 0.01; ***P < 0.001.

We induced sepsis in rats using CLP surgery. Histological analysis showed that the CLP-induced sepsis resulted in pulmonary injury (Figure 2A–2C). Interstitial edema with massive infiltration of inflammatory cells into the interstitial and alveolar spaces was observed, and pulmonary architecture was found to be severely damaged in rats with CLP-induced sepsis. Additionally, the levels of hepatic-injury markers (ALT and AST activities) were significantly higher in the plasma of rats with CLP-induced sepsis as compared to that in control rats (60.2 ± 5.5 vs 16.3 ± 1.85, P < 0.001, and 100.4 ± 6.4 vs 15.8 ± 2.87, P < 0.001, respectively; Figure 2D and 2E). Creatinine and blood urea nitrogen (BUN) concentrations, i.e., markers of renal injury, were also higher in the rats with CLP-induced sepsis than that in the control rats (0.48 ± 0.17 vs 0.11 ± 0.084, P < 0.001, and 81.3 ± 2.46 vs 19.8 ± 3.12, P < 0.001, respectively; Figure 2F and 2G). These data suggested that CLP-induced sepsis causes severe tissue injury, particularly in the lungs.

### Sepsis induces HDAC6 expression, downregulates PHB1, and increases oxidative stress in rats

**Figure 3:**
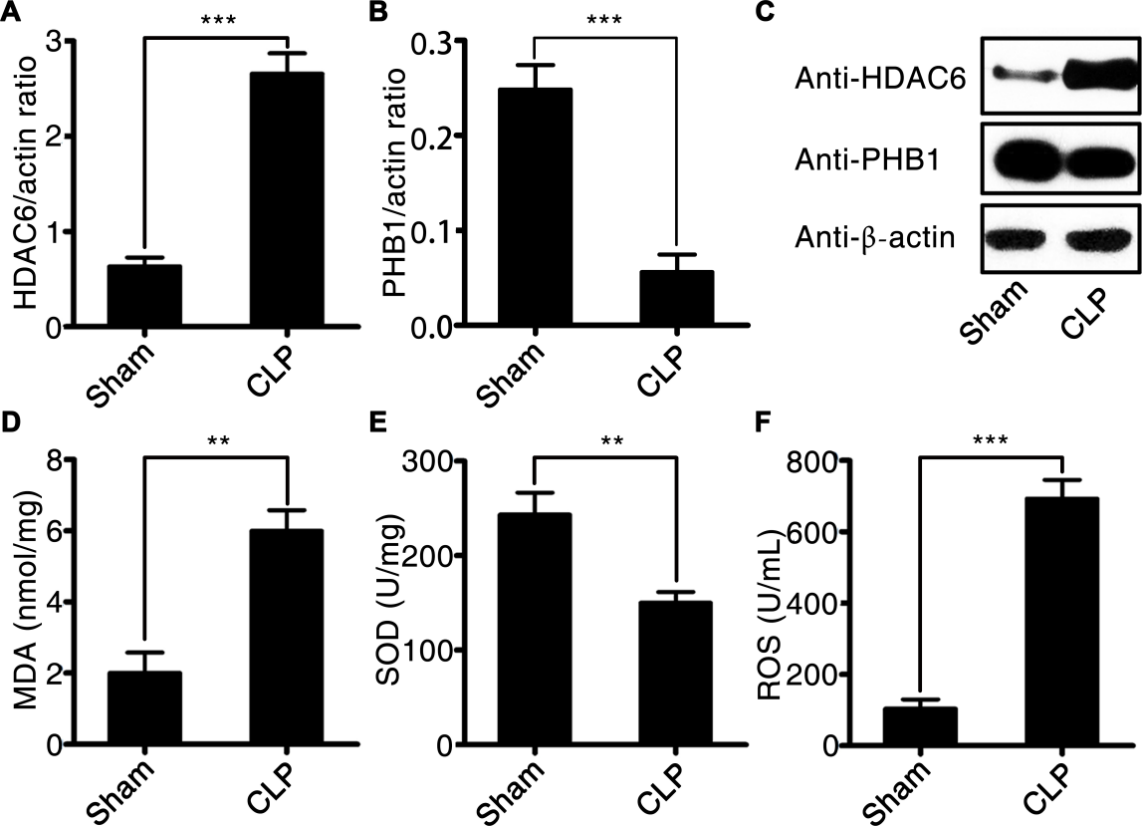
**HDAC6 and PHB1 expression and oxidative stress in rats with CLP-induced sepsis.** (**A**) *HDAC6* mRNA expression; (**B**) *PHB1* mRNA expression; (**C**) HDAC6 and PHB1 protein expression; (**D**) MDA levels; (**E**) SOD activity; and (**F**) ROS production in CLP-induced sepsis rats and healthy control rats. Results are expressed as the mean ± SEM. *P < 0.05; **P < 0.01; ***P < 0.001.

The expression of HDAC6 at mRNA and protein levels was significantly higher in rats with CLP-induced sepsis compared with that in control rats (2.77 ± 0.22 vs 0.71 ± 0.14, P < 0.001). Conversely, the expression of *PHB1* mRNA and the production of PHB1 protein were dramatically lower in the rats with CLP-induced sepsis than that in the control rats (0.055 ± 0.021 vs 0.24 ± 0.032, P < 0.001; Figure 3A–3C). Next, we measured the levels of a tissue injury marker (malondialdehyde; MDA), ROS, and a marker of antioxidant processes (superoxide dismutase; SOD) in rats with CLP-induced sepsis. Sepsis induction was found to be associated with increased levels of MDA and ROS (5.91 ± 0.35 vs 2.02 ± 0.04, P < 0.01; 690.2 ± 45.2 vs 109.3 ± 21.4, P < 0.001, in rats with CLP-induced sepsis vs the control rats, respectively; Figure 3D and 3F). By contrast, SOD activity was dramatically lower in the rats with CLP-induced sepsis than that in the control rats (155.8 ± 18.2 vs 245.1 ± 47.2, P < 0.01; Figure 3E). These findings meant that the expression of HDAC6 and PHB1 is associated with oxidative stress and oxidative injury in different ways during the development of sepsis.

### HDAC6 can regulate PHB1-mediated mitochondrial respiration

**Figure 4:**
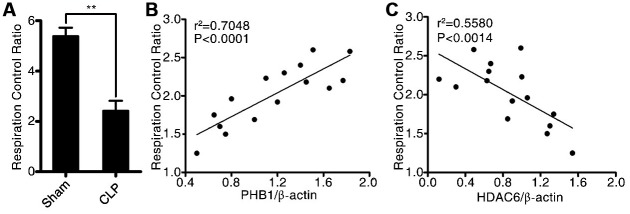
**HDAC6 can regulate PHB1-mediated mitochondrial respiration.** (**A**) The mitochondrial respiratory control rate; (**B**) the correlation between the mitochondrial respiratory control rate and *PHB1* expression; and (**C**) the correlation between the mitochondrial respiratory control rate and *HDAC6* expression in CLP-induced sepsis rats and healthy control rats. Results are expressed as the mean ± SEM. **P < 0.01.

To investigate the potential mechanism by which HDAC6 may promote sepsis development, we first determined the mitochondrial respiratory control rate, and then tested whether it correlated with the expression of HDAC6 and/or PHB1. As depicted in Figure 4A, in rats with CLP-induced sepsis, the mitochondrial respiratory control rate was significantly lower compared to that in the control rats (2.56 ± 0.47 vs 5.25 ± 1.57, P < 0.01). Additionally, we analyzed the linear correlation between HDAC6 and PHB1 expression and the mitochondrial respiratory control rate, and found that in rats with CLP-induced sepsis, the expression of HDAC6 protein negatively correlated with the mitochondrial respiratory control rate (R^2^ = 0.7048, P < 0.0001; Figure 4C) while the expression of PHB1 protein positively correlated with this rate (R^2^ = 0.5580, P < 0.0014; Figure 4B). Taken together, these results indicated that HDAC6 may promote the development of sepsis via regulation of PHB1-mediated mitochondrial function and homeostasis, in vivo.

### HDAC6 inhibits PHB1 expression, thereby resulting in mitochondrial dysfunction

**Figure 5:**
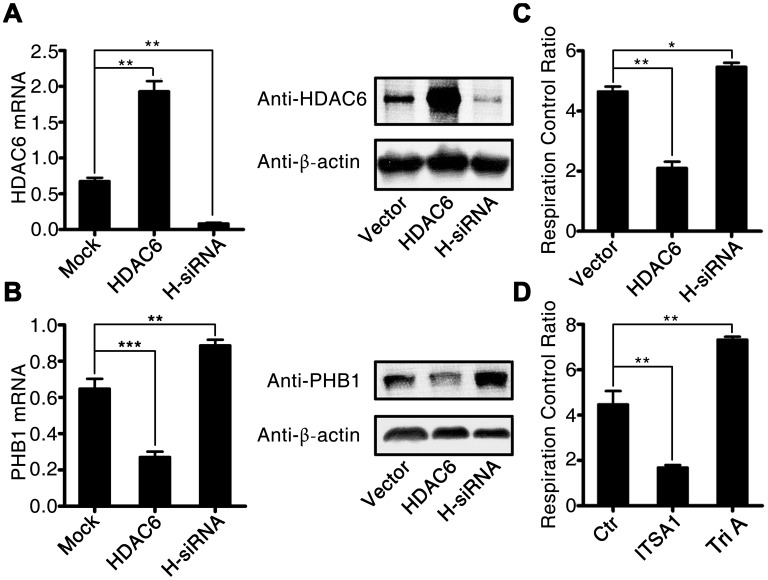
**HDAC6 inhibits the expression of PHB1 and causes subsequent mitochondrial dysfunction.** U937 cells were infected with an *HDAC6*–expressing or HDAC6-specific shRNA–expressing lentivirus, and then gene expression and the mitochondrial respiratory control rate were determined to evaluate the influence of HDAC6 on mitochondrial function. (**A**) HDAC6 mRNA and protein expression; (**B**) PHB1 mRNA and protein expression; and (**C**) the mitochondrial respiratory control rate in lenti-HDAC6 (HDAC6 overexpressing) and lenti-sh-HDAC6 (HDAC6 knockdown) U937 cells. (**D**) The mitochondrial respiratory control rate in HDAC6 agonist (ITSA1) and HDAC6 inhibitor (Tri A) treated macrophages. Results are expressed as the mean ± SEM. **P < 0.01.

To investigate in further detail how HDAC6 promotes oxidative stress during CLP-induced sepsis, we used lentivirus-mediated gene expression and suppression methods to generate HDAC6-overexpressing cells and HDAC6 knockdown U937 cells. The expression of HDAC6 at mRNA and protein levels was significantly higher in the lenti-HDAC6–infected cells (overexpression cell line) and significantly lower in the lenti-sh-HDAC6–infected cells (knockdown via short hairpin RNA [shRNA] expression) as compared to that in uninfected control cells (1.91 ± 0.25 vs 0.75 ± 0.18, P < 0.01, and 0.14 ± 0.034 vs 0.75 ± 0.18, P < 0.01, respectively; Figure 5A). The inhibitory influence of HDAC6 on the expression of PHB1 was also observed in HDAC6-overexpressing cells and in HDAC6 knockdown cells compared to that in the control cells (0.28 ± 0.05 vs 0.65 ± 0.06, P < 0.001, and 0.89 ± 0.07 vs 0.65 ± 0.06, P < 0.01, respectively; Figure 5B). Next, we determined the mitochondrial respiratory control rate in HDAC-overexpressed and HDAC-inhibited cells and found that the overexpression of HDAC6 reduced the mitochondrial respiratory control rate (2.11 ± 0.32 vs 4.63 ± 0.04, P < 0.001), whereas the inhibition of HDAC6 expression increased this rate (5.64 ± 0.03 vs 4.63 ± 0.04, P < 0.05; Figure 5C). To confirm this observation, rat primary macrophages were treated with an HDAC6 agonist (ITSA1) and a selective HDAC6 inhibitor, Tri A (1,2,3,4-tetrahydro-N-hydroxy-2-[(1-methyl-1H-pyrrol-2-yl) carbonyl]-6-isoquinolinecarboxamide). The results of this experiment were consistent with the those obtained from the lentivirus infection assay (Figure 5D).

### HDAC6 inhibition protects against CLP-induced sepsis in rats

**Figure 6:**
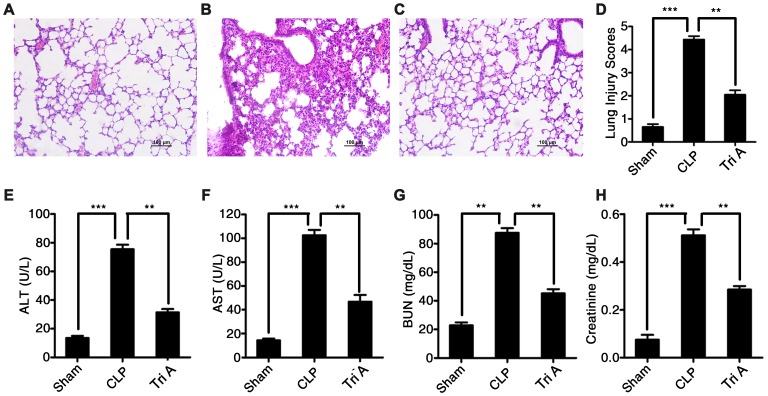
**The impact of HDAC6 inhibition on CLP-induced sepsis.** Hematoxylin and eosin (H&E)-stained lung tissue sections from rats with CLP-induced sepsis, rats with CLP-induced sepsis treated with Tri A, or control rats (×200 magnification). (**A**) Lung tissue from control rats; (**B**) lung tissue from rats with CLP-induced sepsis; (**C**) lung tissue from rats with CLP-induced sepsis treated with Tri A; (**D**) the lung injury index of differentially treated rats; (**E**) ALT activity; (**F**) AST activity; (**G**) creatinine concentration; and (**H**) BUN levels in the plasma from the rats with CLP-induced sepsis, the rats with CLP-induced sepsis treated with Tri A, or the control rats. Results are expressed as the mean ± SEM. *P < 0.05; **P < 0.01; ***P < 0.001.

To determine whether selective inhibition of HDAC6 could attenuate CLP-induced sepsis *in vivo*, we used Tri A (selective HDAC6 inhibitor) to treat the rats with CLP-induced sepsis, and then evaluated possible signs of tissue pathology. Tri A treatment significantly alleviated sepsis-associated lung injury in rats with CLP-induced sepsis in comparison with that in untreated septic rats (Figure 6A–6D). Additionally, plasma ALT activity (Figure 6E), AST activity (Figure 6F), and plasma BUN (Figure 6G) and creatinine levels (Figure 6H) were dramatically lower in the rats with CLP-induced sepsis treated with Tri A relative to that in the untreated septic rats (37.6 ± 1.67 vs 76.5 ± 2.47, P < 0.01; 47.8 ± 2.87 vs 107.2 ± 5.23, P < 0.01; 51.1 ± 2.11 vs 88.7 ± 3.37, P < 0.01; and 0.28 ± 0.02 vs 0.52 ± 0.05, P < 0.01, respectively), indicating that sepsis-induced hepatic and renal injuries were attenuated by HDAC6 inhibition. Taken together, these data implied that HDAC6 may serve as a target in the treatment of sepsis.

## DISCUSSION

Oxidative stress is associated with the severity of sepsis, and the level of oxidative stress is determined by mitochondrial respiratory function [6]. In the present study, we found that the expression of HDAC6 was significantly higher in PBMCs from sepsis patients than in PBMCs from healthy control participants. Mechanistic experiments revealed that HDAC6 can inhibit the expression of PHB1, and can subsequently enhance some activities of mitochondrial respiratory function (which are suppressed by PHB1) *in vivo*, thereby promoting the production of oxidants and oxidative injury.

In most of eukaryotic cells, mitochondria provide energy by generating adenosine triphosphate (ATP) through oxidative phosphorylation and by controlling oxidative stress [28]. During normal functioning of the mitochondrial respiratory chain, ROS such as superoxide radical (O_2_^−^), hydrogen peroxide (H_2_O_2_), and hydroxyl radicals (OH·) are generated in low amounts. As a mitochondrial protein, PHB1 plays an important role in cell proliferation, apoptosis, gene transcription [29–31], and protein folding [32]. Additionally, PHB1 has been demonstrated to keep the mitochondrial respiratory chain in check [33]. PHB1 knockdown in endothelial cells increases the production of ROS in mitochondria via decreased activity of complex I and partial blockade of the electron transport chain [34]. Overexpression of PHB1 in cultured cardiomyocytes protects the mitochondria from injury, prevents hydrogen peroxide-induced apoptosis of cardiomyocytes, and maintains mitochondrial membrane permeability [35]. Herein, we found that PHB1 expression is significantly lower in PBMCs from sepsis patients than that in PBMCs derived from healthy controls. We also noticed that the expression of HDAC6 was dramatically higher in PBMCs derived from sepsis patients than that in PBMCs from healthy control participants. Of note, when we analyzed the correlation between HDAC6 and PHB1 in PBMCs from sepsis patients, we found a negative linear correlation between the expression of HDAC6 and PHB1, meaning that the expression of PHB1 may be downregulated by HDAC6 during the development of sepsis.

To further understand the effects of HDAC6 during sepsis development, we created a rat model of CLP-induced sepsis. The CLP-induced sepsis in rats was associated with severe pulmonary, hepatic, and renal injury. We also observed that HDAC6 expression was higher while PHB1 expression was lower in the rats with CLP-induced sepsis compared to that in rats in the control group; this was consistent with the data obtained from human sepsis patients. Further investigation revealed that HDAC inhibited PHB1 expression and subsequently altered the efficiency of the mitochondrial respiratory chain, thereby increasing the production of ROS, resulting in the observed oxidative injury to multiple organ tissues.

To evaluate whether HDAC6 could serve as a therapeutic target in sepsis, we treated rats with CLP-induced sepsis with a selective HDAC6 inhibitor (Tri A). HDAC6 inhibition significantly attenuated the sepsis-associated pulmonary, hepatic, and renal injury, suggesting that inhibition of HDAC6 may form the basis of a potential therapy for sepsis and related disorders. Nonetheless, there are several limitations of this study. First, the mechanism by which HDAC6 reduces the expression of PHB1 needs to be further investigated. Second, we did not confirm that PHB2 is involved in the process. Third, broad-spectrum antibiotic administration is considered to be the standard of care for patients with sepsis, but whether this treatment affects the expression of HDAC6 and PHB1 needs to be studied.

In summary, we found that a histone deacetylase, HDAC6, inhibited the expression and function of PHB1, a mitochondrial protein. The HDAC6-induced downregulation of PHB1 subsequently altered the functioning of the mitochondrial respiratory chain, enhanced the production of oxidants, and increased oxidative stress, leading to severe oxidative injury in multiple tissues. Inhibition of HDAC6 activity significantly reduced the multiple organ injury caused by oxidative stress *in vivo.*

## MATERIALS AND METHODS

### Patients and tissue samples

Sepsis patients and healthy controls were selected as the subjects of this study in China-Japanese Friendship Hospital, and the criteria for sepsis diagnosis were as follows. For infected patients or patients with suspected infection, when the score of sepsis-related sequential organ failure (SOFA) increased by more than 2 points (contains 2 points), sepsis could be diagnosed. We collected 4 mL of venous blood from all the subjects on the day of admission. PBMCs were isolated and stored in liquid nitrogen until use. Approval was obtained from the Institutional Ethics Committee of The China-Japan Friendship Hospital; moreover, informed consent was obtained from all the patients before the study was initiated.

There was no significant difference in demographic characteristics between the two groups (P > 0.05; Table 1). The clinical characteristics of sepsis patients are presented in Table 2.

**Table 1:** Demographic characteristics of the two groups.

	**sepsis patient (n=95)**	**healthy control (n=95)**	**t/χ^2^**	**P**
Age (year)	68.5 ± 7.3	67.6 ± 5.9	3.612	0.267
Body mass index(kg/m2)	21.27 ± 3.56	21.59 ± 2.85	2.159	0.315
Male n (%)	31 (62.0)	34 (68.0)	0.791	0.374
With underlying disease n (%)	27 (54.0)	29 (58.0)	0.325	0.569
Without underlying disease n (%)	23 (46.0)	21 (42.0)		

**Table 2:** Clinical characteristics of sepsis patients.

	**sepsis patient (n=95)**
Heart rate (beats/min)	115.6 ± 17.3
Temperature (°C)	38.62 ± 0.93
Pa_O2_/F_IO2_	169.53 ± 16.71
SOFA score	6.5 ± 2.8
APACHE II score	11.3 ± 5.2
Positive blood culture n (%)	24 (48.0%)
Lactate (mmol/L)	4.71 ± 1.35
C-reactive protein (mg/L)	89.65 ± 16.23
Leucocytes (10^9^ cells/L)	14.26 ± 3.75
fasting blood-glucose (mmol/L)	8.6 ± 2.1

### Antibodies and reagents

A rabbit anti-HDAC6 antibody was purchased from Abcam (Cambridge, UK; cat. # ab1440; 1:1000), whereas a rabbit anti-PHB1 antibody (#2426, 1:1000), rabbit anti-PHB2 antibody (#14085, 1:1000), and rabbit anti-β-actin antibody (#4967, 1:1000) were bought from Cell Signaling Technology (Danvers, MA, USA). Tri A was acquired from Cayman Chemical (Item No. 15200). The HDAC activator, ITSA1, was purchased from MedChemExpress (HY-100508).

### Animals

Healthy male Sprague–Dawley rats (8 weeks old, 200–220 g, n = 45) were bought from Shanghai Model Organisms Center (Shanghai, China). All the rats were housed in a specific pathogen-free laboratory animal room, fed a standard chow diet, and maintained on a 12 h light/dark cycle at 24 ± 2°C and 40–70% humidity. The rats were allowed free access to water and feed. All animal experiments were conducted in accordance with the National Institutes of Health Guide for the Care and Use of Laboratory Animals and were approved by the Ethics Committee of China-Japan Friend Hospital (approval No. 2018-49-K38).

Rats were randomized into three groups (n = 15 per group): (i) mock/control group, (ii) CLP-induced sepsis group, and (iii) CLP-induced sepsis + HDAC6 inhibitor treatment (Tri A, 2 mg/kg) group.

### The CLP-induced sepsis model

The rat model of sepsis was generated by CLP, as described previously [36, 37]. Briefly, after 12 h fasting, rats were anesthetized with 2% pentobarbital, and a midline incision was made along the linea alba of the abdominal muscle to isolate and expose the cecum. The latter was ligated and then punctured twice through the center of the ligated segment using an 18-gauge needle. Any remaining feces were gently extruded from the punctures to ensure patency. The cecum was returned to the peritoneal cavity, and the abdominal incision was sutured. Rats in the control group were anesthetized and received a midline incision, as described above. In contrast, the cecum was neither ligated nor punctured in the control group.

Rats were given fluids (50 mL/kg 0.9% normal saline, subcutaneously) at the time of the surgical procedure and every 6 h thereafter and antibiotics (ceftriaxone 30 mg/kg and clindamycin 30 mg/kg, intramuscularly) every 6 h. Immediately after the CLP procedure, rats in the HDAC6 inhibition group received an intraperitoneal injection of Tri A. Rats in the control group received an equal volume of saline. At 24 h postsurgery, five rats in each group were randomly selected and euthanized. Blood was collected from the femoral vein. The remaining 10 rats in each group were examined twice a day for 4 days, and survival was documented.

### Histopathology

Lung tissues were collected 24 h after CLP. Tissues were fixed in 4% formaldehyde for 24 h at 4°C. Samples were then embedded in paraffin and sectioned (5 μm) and stained with hematoxylin and eosin. The stained sections were observed under a light microscope (Olympus BX51, Olympus, Tokyo, Japan) and evaluated by three independent pathologists. The stained sections were given a lung injury score from 0 to 4, with 0 indicating no injury, 1 denoting modest injury (limited congestion and interstitial edema, no interstitial neutrophilic infiltrate or inflammatory cells in alveolar spaces), 2 meaning intermediate injury (mild congestion, interstitial edema, and interstitial neutrophilic infiltrate and inflammatory cells in alveolar spaces), 3 indicating widespread injury (prominent congestion and interstitial edema with neutrophils partially filling the alveolar spaces), and 4 representing widespread injury (marked congestion, interstitial edema with the neutrophilic infiltrate nearly filling the alveolar spaces or with lung consolidation). Averages of the lung injury scores served as a semiquantitative histological index of lung injury.

### Cell lines and cultures

The human U937 cell line was purchased from the American Type Culture Collection (ATCC, Manassas, VA, USA). The cells were cultured in the RPMI 1640 medium supplemented with 10% of fetal bovine serum (FBS; Gibco) in a humidified atmosphere at 37°C and 5% CO_2_.

### Establishment of HDAC6 knockdown cells and HDAC6-overexpressing cells

As described previously [38], the knockdown of HDAC6 in U937 cells (lenti-sh-HDAC6) was performed using MISSION® shRNA Lentiviral Transduction Particles (Sigma-Aldrich, St. Louis, MO, USA). The target sequence was 5′- CCGGCCTCACTGATCAGGCCATATTCTCGAGAATATGGCCTGATCAGTGAGGTTTTT-3′. HDAC6-overexpressing U937 cells (lenti-HDAC6) were generated by means of human HDAC6 Lentivirus (pLenti-GIII-UbC, LVP710809, ABM Inc., USA). Lentiviral particles (200 μL in 4 mL of the RPMI 1640 medium supplemented with 10% of FBS) were added to cell culture media and were incubated with the cells for 24 h at 37°C and 5% CO_2_. Successfully infected cells (lenti-sh-HDAC6 or lenti-HDAC6) were selected by culturing the transduced cells in the presence of puromycin (3 μg/mL in the medium with 10% of FBS) for 48 h. The cells were then harvested, and the expression of HDAC6 was determined by quantitative PCR (qPCR) and western blotting.

### qPCR

As described previously [39], total RNA was extracted from tissues or cells using the TRIzol reagent (Invitrogen, Carlsbad, CA, USA) according to the manufacturer’s instructions. cDNA was synthesized with the High-Capacity cDNA Reverse Transcription Kit (Applied Biosystems, Foster City, CA, USA). qPCR was conducted using the SYBR Mastermix following the manufacturer’s instructions (Applied Biosystems). All the reactions were carried out on a 7900 Real-time PCR System (Applied Biosystems). β-Actin served as a control for normalization. Relative expression levels of target genes were calculated by the 2^ΔC(t)^ method. The following primer sequences were employed: HDAC6, 5′-CTCATCCTGCGCTGTCTGGT-3′ (forward), 5′-CCGGAGCTGCCTGACCTCGG-3′ (reverse); PHB1, 5′-ATCGCTATGGCATTCCATG-3′ (forward), 5′-AATTCGATGCAATTGCCGTA-3′ (reverse); and β-actin, 5′-GTCTCCTCTGACTTCAACAGCG-3′ (forward), 5′-ACCACCCTGTTGCTGTAGCCAA-3′ (reverse). Gene expression was presented as the HDAC6/β-actin or PHB1/β-actin ratio and was calculated as follows: ratio = 2^(ΔCt[β-actin] − ΔCt[Target gene])^.

### Western blot

As described elsewhere [40], cell and tissue samples were lysed in RIPA buffer, sonicated on ice, and centrifuged at 200 × *g* for 30 min at 4°C. The samples were then boiled in sodium dodecyl sulfate polyacrylamide gel electrophoresis (SDS-PAGE) loading buffer for 10 min and subjected to SDS-PAGE in a 10% gel, before transfer onto nitrocellulose membranes (Millipore, Billerica, MA, USA). The membranes were probed with primary antibodies overnight at 4°C and then with horseradish peroxidase–conjugated secondary antibodies. Bands were visualized by means of an Enhanced Chemiluminescence Substrate (GE Healthcare, Marlborough, MA, USA).

### Detection of organ injury markers

Plasma levels of creatinine and BUN were measured using a commercial assay kit (Pointe Scientific, Lincoln Park, MI) according to the manufacturer’s instructions. ALT and AST activities were measured on a Hitachi 7600-020 Automatic Analyzer (Hitachi High-Technologies Corporation, Tokyo, Japan).

### Measurement of MDA concentration and SOD activity

MDA levels and SOD activity were determined by means of the Thiobarbituric Acid Assay Kit (Solarbio, Beijing, China) and a Xanthine and Xanthine Oxidase Reaction System (Abcam, Cambridge, UK), respectively, according to the manufacturers’ protocols. Absorbance was read at 532 nm (MDA) and 450 nm (SOD) on a microplate reader (Bio-Tek, Shanghai, China). The results were calculated according to the manufacturer’s instructions and were normalized to total protein concentration.

### Quantitation of ROS

Serum levels of ROS were determined with the ROS-Glo^TM^ Assay Kit (Promega, Madison, WI, USA) according to the manufacturer’s instructions.

### Measurement of the mitochondrial respiratory rate

Mitochondrial respiratory rate was measured in saponin-permeabilized cardiac fibers by high-resolution respirometry as described previously [41]. Briefly, respiration was quantified following the addition of glutamate (10 mM) and malate (2 mM) as complex I substrate supply (V_0_). VO_2max_ was measured after the addition of 2.5 mM ADP, and V_oligo_ was measured following the addition of 1 μg/mL oligomycin. O_2_ flux was calculated from the negative time derivative of the oxygen concentration signal using DatLab 4 software (Oroboros Instruments, Innsbruck, Austria). Respiration results were normalized to fiber weight to adjust for potential differences in the mitochondrial content.

### Statistical analysis

All results were expressed as the mean ± standard error of the mean (SEM). The analyses were performed using GraphPad Prism (GraphPad Software, La Jolla, CA, USA). The Student *t* test (two-tailed) and one-way analysis of variance (ANOVA) were conducted to compare various experimental groups. When more than two groups were included in an analysis, Bonferroni’s correction of the significance level was applied. Correlations among the expressions of HDAC6 and PHB1 and the mitochondrial respiratory control rate were assessed via Spearman’s correlation coefficients. Data with a p value less than 0.05 were considered statistically significant.

### Ethics approval

All procedures in the experiments involving human participants and animals were conducted in accordance with the ethical standards of The China-Japan Friendship Hospital. Informed consent to participate in the study was obtained from all the participants.
